# Delirium in elderly patients with COPD combined with respiratory failure undergoing mechanical ventilation: a prospective cohort study

**DOI:** 10.1186/s12890-022-02052-5

**Published:** 2022-07-09

**Authors:** Xuecai Fu, Lina Wang, Guihua Wang, Xuefang Liu, Xin Wang, Shuiting Ma, Fengru Miao

**Affiliations:** Geriatrics Department, Aerospace Center Hospital, No 15 Yuquan Road, Haidian District, Beijing, China

**Keywords:** COPD, Respiratory failure, Mechanical ventilation, Elder, Delirium, Care, Management, Prevention

## Abstract

**Background:**

COPD combined with respiratory failure is very common in intensive care unit (ICU). We aimed to evaluate the current status and influencing factors of delirium in elderly COPD patients with undergoing mechanical ventilation.

**Methods:**

Patients with COPD combined with respiratory failure and mechanically ventilated who were admitted to the ICU of our hospital were selected. The characteristics of included patients were assessed. Pearson correlation analysis was performed to evaluate the characteristics of patients and delirium. Logistic regression analysis was conducted to identify the risk factors of delirium in elderly patients with COPD combined with respiratory failure undergoing mechanical ventilation.

**Results:**

A total of 237 COPD combined with respiratory failure patients were included, the incidence of delirium was 21.94%. Pearson correlation analysis indicated that age (r = 0.784), BMI (r = 0.709), hypertension (r = 0.696), APACHE II score (r = 0.801), CPOT (r = 0.513), sedation(r = 0.626) and PaO_2_ (r = 0.611) were all correlated with the occurrence of delirium (all p < 0.05). Logistic regression analysis indicated that age ≥ 75y (OR 3.112, 95% CI 2.144–4.602), BMI ≤ 19 kg/m^2^ (OR 2.742, 95% CI 1.801–3.355), hypertension(OR 1.909, 95% CI 1.415–2.421), APACHE II score ≥ 15 (OR 2.087, 95% CI 1.724–2.615), CPOT ≥ 5 (OR 1.778, 95% CI 1.206–2.641), sedation(OR 3.147, 95% CI 2.714–3.758), PaO_2_ ≤ 75 mmHg(OR 2.196, 95%CI 1.875–3.088) were the risk factors of delirium in elderly patients with COPD combined with respiratory failure undergoing mechanical ventilation (all p < 0.05).

**Conclusions:**

Delirium is common in patients with COPD and respiratory failure undergoing mechanical ventilation, and there are many related influencing factors. Medical staff should pay more attention to patients with risk factors and take intervention measures as soon as possible to reduce the incidence of delirium.

## Background

The incidence of respiratory failure is increasing year by year, and the respiratory failure caused by chronic obstructive pulmonary disease (COPD) is particularly significant. COPD patients often have the characteristics of poor basic pulmonary function, long course of disease, easy relapse, and multiple system diseases [1]. Respiratory failure is the very common reason that people admitted to the intensive care unit (ICU) [2]. Previous studies [3–5] have shown that mechanical ventilation is an effective mean to treat respiratory failure, and it has significant effect on the treatment of patients with COPD and respiratory failure. Patients undergoing mechanical ventilation may have a series of psychological reactions, leading to changes in cognitive function or perceptual impairment and delirium in the ICU [6, 7]. The results of previous studies [8–10] have shown that the incidence of delirium in mechanically ventilated patients can be as high as 60.48%. Once delirium occurs, patients may experience prolonged hospital stay, difficulty in weaning mechanical ventilation, cognitive dysfunction, increased mortality, etc. [11, 12].

Delirium is a common yet easily overlooked abnormal mental state in elderly patients. It is manifested as a series of symptoms characterized by varying degrees of cognitive impairment, lack of orientation, and confusion, forming a syndrome [13]. Delirium is often characterized by obvious cognitive dysfunction and changes in the level of consciousness as the basic clinical features [14]. Some patients may also be accompanied by abnormalities in orientation, attention, perception, and sleep cycles [15]. The prevention and early nursing care of delirium is vital to the prognosis of respiratory failure patients undergoing mechanical ventilation [16, 17]. Currently, the risk factors of delirium in elderly patients with COPD combined with respiratory failure undergoing mechanical ventilation remain unclear. Therefore, we aimed to evaluate the characteristics of elderly patients with COPD combined with respiratory failure undergoing mechanical ventilation, to identify the risk factors of delirium in respiratory failure patients undergoing mechanical ventilation, to provide insights to the clinical management and nursing care of COPD.

## Methods

### Ethics

In this study, all methods were performed in accordance with the relevant guidelines and regulations. This present study was a prospective cohort design. The study protocol had been checked and approved the ethical committee of our hospital (approval number: IC19060048). Besides, the informed consents had been obtained from all the relatives of included patients.

### Patients

In this study, the patients undergoing mechanic ventilation secondary to acute exacerbation of COPD admitted to the ICU of our hospital were selected as the research population. The inclusion criteria for patients were: ① The age of patient was ≥ 65 years; ② Patients diagnosed as COPD complicated with respiratory failure; ③ Patients received mechanical ventilation for more than 24 h. The exclusion criteria for patients are: ① Patients with a history of mental illness or impaired consciousness with Glasgow Coma Scale (GCS) score ≤ 6 at admission to hospital; ②patients treated with non-invasive ventilation (NIV) were also excluded; ③ Patients who actually died during the hospital stay since the duration of mechanical ventilation can obviously be biased by mortality; Patients who were unwilling to participate in the study by themselves or their family members.

### Calculation of sample size

The sample size of this study was calculated according to the comparison formula of two groups[18, 19]: $n = \frac{\lambda }{{2\left( {\arcsin \sqrt {P_{\max } - \arcsin \sqrt P_{\min } } } \right)^{2} }}$, we presumed that α = 0.05, β = 0.1, ν = 3 − 1 = 2, λ = 8.86, we set Pmax, Pmin, respectively, as the maximum and minimum rate of delirium in COPD patients (38.26% and 6.02%)[20], then it comes to the results that n≈182. Meanwhile, considering the potential loss rate of about 10% patients, a total of 201 patients shall be included. We attempted to included more patients as much as possible.

### Diagnosis of delirium

In this study, the Confusion Assessmente Method in the Intensive Care Unit [21] was used to evaluate patients for delirium for the entire ICU stay. CAM-ICU included 4 aspects: ① sudden changes or fluctuations in mental state, ② inattention, ③ changes in level of consciousness, ④ disordered thinking. When patients met both ① and ②, and ③ or ④, the patient could be diagnosed as having delirium. We evaluate the patient for delirium at 8:00, 16:00, and 22:00 every day.

### Data collection

Before the start of the study, we conducted a unified training for the investigators, and explained the purpose and details of the study to the patients and their families to obtain cooperation. We collected clinical data of patients by asking their family members, consulting medical records and using scales. We collected that data including age, gender, BMI, hypertension, diabetes mellitus, hyperlipidemia, anemia, Acute Physiology and Chronic Health Score (APACHE II), Critical Pain Observation Tool (CPOT) [22], use of sedation, duration of mechanical ventilation. CPOT was developed for ICU patients who are unable to communicate. The CPOT scale was used to evaluate patient’s pain status at 8:00 daily, including facial expressions, physical activity, muscle tension, and compliance with mechanical ventilation four items. Each item was rated as 0 to 2 points, and the total score varied from 0 to 8 points. The higher the score, the more serious the patient's pain.

Besides, we collected the laboratory examination results before mechanical ventilation, including: levels of albumin, hemoglobin, blood sugar, creatinine, urea nitrogen, triiodothyronine(T3), thyroxine(T4), thyroid stimulating hormone (TSH), free triiodothyronine (FT3), free thyroxine(FT4), arterial partial pressure of oxygen (PaO_2_), arterial partial pressure of carbon dioxide(PaCO_2_).

### Statistical methods

In this study, SPSS 22.0 statistical software was used for data analysis. Enumeration data were expressed as a percentage, and comparison between groups was performed by chi-square test. Measurement data were expressed as mean ± standard deviation, and comparison between groups was performed by t test. Pearson correlation analysis was performed to evaluate the characteristics of patients and delirium. Logistic regression analysis was conducted to identify the risk factors of delirium in elderly patients with COPD combined with respiratory failure undergoing mechanical ventilation. The difference between groups was statistically significant with P < 0.05.

## Results

### The characteristics of included COPD patients

A total of 237 COPD combined with respiratory failure patients were included, of whom 52 patients had the occurrence of delirium, the incidence of delirium was 21.94%. As indicated in Table 1, there were significant differences in the age, BMI, hypertension, APACHE II score, CPOT, sedation, duration of mechanical ventilation between delirium and no delirium group (all p < 0.05). No significant differences in the gender, diabetes mellitus, hyperlipidemia, anemia between delirium and no delirium group were found (all p > 0.05).

**Table 1:** The characteristics of included COPD patients

Variables	Delirium group(n = 52)	No delirium group(n = 185)	t/χ^2^	p
Age(y)	78.11 ± 10.26	69.84 ± 9.03	9.445	0.016
Male/female	31/21	110/75	2.061	0.093
BMI (kg/m^2^)	18.12 ± 4.67	20.05 ± 4.19	3.142	0.007
Hypertension	40(76.92%)	66(35.68%)	1.065	0.024
Diabetes mellitus	25(48.08%)	84(45.41%)	3.129	0.108
Hyperlipidemia	20(38.46%)	67(36.22%)	2.025	0.082
Anemia	28(53.85%)	92(49.73%)	1.113	0.104
APACHE II score	17.49 ± 4.07	12.62 ± 3.28	5.108	0.008
CPOT	6.14 ± 2.01	4.21 ± 1.66	2.045	0.015
Sedation	41(78.85%)	59(31.89%)	1.251	0.002
Duration of mechanical Ventilation(h)	73.14 ± 22.17	68.05 ± 23.14	11.038	0.048

*BMI* Body mass index, *APACHE II* acute physiology and chronic health score, *CPOT* critical pain observation tool

The laboratory examination results before mechanical ventilation.

As indicated in Table 2, there were significant difference in the PaO_2_ between delirium and no delirium group (p = 0.016). No significant differences in the albumin, hemoglobin, blood sugar, creatinine, urea nitrogen, pH, T3, T4, TSH, FT3, FT4 and PaCO_2_ were found (all p > 0.05).

**Table 2:** The laboratory examination results before mechanical ventilation

Variables	Delirium group(n = 52)	No delirium group(n = 185)	t/χ^2^	p
Albumin(g/L)	41.91 ± 8.02	42.88 ± 10.24	6.132	0.081
Hemoglobin(g/L)	123.63 ± 17.45	125.12 ± 13.44	13.562	0.103
Blood sugar(mmol/L)	7.46 ± 1.27	7.51 ± 1.35	1.241	0.196
Creatinine(μmol/L)	76.53 ± 9.82	74.17 ± 8.95	5.033	0.094
Urea Nitrogen(mmol/L)	7.72 ± 1.25	7.49 ± 1.47	2.204	0.176
pH	7.38 ± 1.15	7.40 ± 1.02	3.847	0.113
T3(mU/L)	1.42 ± 0.35	1.36 ± 0.28	1.012	0.095
T4(mU/L)	96.05 ± 12.15	97.24 ± 14.02	4.225	0.061
TSH(mU/L)	2.42 ± 0.35	2.63 ± 0.53	1.607	0.084
FT3(mU/L)	3.18 ± 0.86	3.26 ± 0.95	1.874	0.262
FT4(mU/L)	16.94 ± 5.64	16.66 ± 5.13	2.084	0.114
PaO_2_(mmHg)	71.21 ± 19.05	79.41 ± 15.89	10.123	0.016
PaCO_2_(mmHg)	34.01 ± 7.24	32.54 ± 8.38	5.019	0.052

*T3* Triiodothyronine, *T4* thyroxine; *TSH* thyroid stimulating hormone, *FT3* free triiodothyronine, *FT4* free thyroxine, *PaO*_*2*_ arterial partial pressure of oxygen, *PaCO*_*2*_ arterial partial pressure of carbon dioxide

### Pearson correlation analysis

As showed in Table 3, Pearson correlation analysis indicated that age(r = 0.784), BMI(r = 0.709), hypertension(r = 0.696), APACHE II score(r = 0.801), CPOT(r = 0.513), sedation(r = 0.626) and PaO_2_ (r = 0.611) were all correlated with the occurrence of delirium (all p < 0.05).

**Table 3:** Pearson correlation analysis on the characteristics of patients and delirium

Variables	r	P
Age(y)	0.784	0.006
Gender	0.016	0.112
BMI (kg/m^2^)	0.709	0.014
Hypertension	0.696	0.045
Diabetes mellitus	0.123	0.079
Hyperlipidemia	0.126	0.205
Anemia	0.165	0.148
APACHE II score	0.801	0.025
CPOT	0.513	0.036
Sedation	0.626	0.029
Duration of mechanical ventilation(h)	0.265	0.103
Albumin(g/L)	0.163	0.088
Hemoglobin(g/L)	0.124	0.105
Blood sugar(mmol/L)	0.168	0.059
Creatinine(μmol/L)	0.042	0.146
Urea nitrogen(mmol/L)	0.146	0.093
T3(mU/L)	0.113	0.119
T4(mU/L)	0.147	0.101
TSH(mU/L)	0.073	0.162
FT3(mU/L)	0.187	0.067
FT4(mU/L)	0.153	0.122
PaO_2_(mmHg)	0.611	0.021
PaCO_2_(mmHg)	0.195	0.089

*BMI* Body mass index, *APACHE II* acute physiology and chronic health score, *CPOT* critical pain observation tool, *PaO*_*2*_ arterial partial pressure of oxygen, *T3* Triiodothyronine, *T4* thyroxine; *TSH* thyroid stimulating hormone, *FT3* free triiodothyronine, *FT4* free thyroxine, *PaO*_*2*_ arterial partial pressure of oxygen, *PaCO*_*2*_ arterial partial pressure of carbon dioxide

### Logistic regression analysis

The variable assignment of multivariate logistic regression was indicated in Table 4. As shown in Table 5, Logistic regression analysis indicated that age ≥ 75y(OR 3.112, 95% CI 2.144–4.602), BMI ≤ 19 kg/m^2^(OR 2.742, 95% CI 1.801–3.355), hypertension(OR 1.909, 95% CI 1.415–2.421), APACHE II score ≥ 15(OR 2.087, 95% CI 1.724–2.615), CPOT ≥ 5(OR 1.778, 95% CI 1.206–2.641), sedation(OR 3.147, 95% CI 2.714–3.758), PaO_2_ ≤ 75 mmHg(OR 2.196, 95%CI 1.875–3.088) were the risk factors of delirium in elderly patients with COPD combined with respiratory failure undergoing mechanical ventilation(all p < 0.05).

**Table 4:** The variable assignment of multivariate logistic regression

Factors	Variables	Assignment
Delirium	Y	Yes = 1, no = 2
Age(y)	X_1_	≥ 75y = 1, < 75y = 2
BMI(kg/m^2^)	X_2_	≤ 19 = 1, > 19 = 2
Hypertension	X_3_	Yes = 1, no = 2
APACHE II score	X_4_	≥ 15 = 1, < 15 = 2
CPOT	X_5_	≥ 5 = 1, < 5 = 2
Sedation	X_6_	Yes = 1, no = 2
PaO_2_(mmHg)	X_7_	≤ 75 = 1, > 75 = 2

*BMI* Body mass index, *APACHE II* acute physiology and chronic health score, *CPOT* critical pain observation tool, *PaO*_*2*_ arterial partial pressure of oxygen

**Table 5:** Logistic regression analysis on the risk factors of delirium in elderly patients with COPD combined with respiratory failure undergoing mechanical ventilation

Items	β	SE	OR	95%CI	p
Age ≥ 75y	0.113	0.201	3.112	2.144–4.602	0.016
BMI ≤ 19 kg/m^2^	0.215	0.316	2.742	1.801–3.355	0.024
Hypertension	0.164	0.293	1.909	1.415–2.421	0.032
APACHE II score ≥ 15	0.286	0.541	2.087	1.724–2.615	0.007
CPOT ≥ 5	0.195	0.426	1.778	1.206– 2.641	0.023
Sedation	0.167	0.541	3.147	2.714 – 3.758	0.017
PaO_2_ ≤ 75 mmHg	0.118	0.377	2.196	1.875–3.088	0.009

*BMI* body mass index, *APACHE II* Acute Physiology and Chronic Health Score, *CPOT* Critical Pain Observation Tool, *PaO*_*2*_ arterial partial pressure of oxygen

## Discussions

Respiratory failure patients often have multiple complications due to severe disease and rapid development, and their risk of delirium is particularly high [23, 24]. In recent years, researches on the delirium in the ICU have gradually deepened and refined. The incidence of delirium in the ICU was 10.13–78.46%, of which mechanically ventilated patients were the majority [25, 26]. At present, there is still a lack of studies on the current status of delirium in patients with COPD and respiratory failure undergoing mechanical ventilation [27]. The results of this study have indicated that the incidence of delirium in patients with COPD combined with respiratory failure and mechanically ventilated is 21.94%, and for patients with age ≥ 75y, BMI ≤ 19 kg/m^2^, hypertension, APACHE II score ≥ 15, CPOT ≥ 5, sedation, PaO_2_ ≤ 75 mmHg are the risk factors of delirium in elderly patients with COPD combined with respiratory failure undergoing mechanical ventilation, early targeted intervention and nursing care are needed for those patients to reduce the occurrence of delirium.

Advanced age is an independent risk factor for delirium. COPD patients are mostly elderly patients. The cause of delirium is the abnormal function of neurotransmitter and the damage of blood–brain barrier [28–30]. The receptivity of the brain of the elderly to external stimuli decreases, and the inflammation response increases, which is manifested by the progressive apoptosis of the cells in the brain of the elderly, the decline of brain function, and the decrease of the activity of neurotransmitter synthase in the brain with aging [31, 32]. In addition, the activation of microglia in the brain of elderly patients is more obvious, the neuron microenvironment changes, and the level of inflammatory factors is increased. Under the stimulation of mechanical ventilation, it can produce a stronger and longer lasting inflammatory response, which can cause to the abnormalities of neurotransmitters in the brain and damage to the blood–brain barrier, leading to the occurrence of delirium [33, 34].

The results of this study found that BMI lower than 19 kg/m^2^ is an important factor for delirium in elderly patients with COPD complicated with respiratory failure. A number of previous studies [35–37] have shown that BMI < 20 kg/m^2^ is a risk factor for postoperative delirium, which is consistent with our findings. Lower BMI is associated with the poor nutrition status [38]. For patients with poor nutritional status, nutritional support treatment is recommended before elective surgery, actively correcting hypoproteinemia and adjusting nutritional status, which may help reduce the occurrence of postoperative delirium [39, 40].

The APACHE II score represents the severity of the disease, and the score is positively correlated with the severity of the disease. The higher the APACHE II score of the patient, the more severe the stress response caused by the disease, the more obvious the acute disorder of high-level nerve center activity, and the more prone to delirium [41]. Therefore, while paying attention to disease treatment, medical staff should comprehensively evaluate the patient's condition, closely observe the patient's consciousness, blood oxygen saturation, disease manifestations, laboratory indicators, etc., and take timely measures [42]. In addition, predictive assessment is particularly important to effectively predict the potential risks of such patients, and take immediate measures to avoid deterioration of the disease [43].

Patients with COPD combined with respiratory failure often have a decrease in PaO_2_. The lower the PaO_2_, the more severe the patient’s hypoxia and the more obvious the internal environment disorder. The oxygen supply of the brain tissue of COPD patients decreases [44]. The lower the PaO_2_, the more obvious the imbalance on the oxygen supply and the more serious the brain tissue edema [45]. In addition, the lower the PaO_2_, the more obvious the symptoms of shortness of breath and wheezing, and the stronger the general discomfort, which is often accompanied by profuse sweating, decreased blood pressure, rapid heart rate, etc., and the patient is prone to negative psychology such as anxiety, fear, irritability, and even mental behavior, which in turn induces delirium [46, 47]. Studies [48, 49] have pointed out that patients with reduced oxygen saturation and hypoxemia are more likely to develop delirium. Therefore, medical staff should pay more attention to patients with PaO_2_ < 75 mmHg, and be particularly vigilant in patients with tissue hypoperfusion. If conditions permit, the medical stuff should remove the tracheal intubation as soon as possible, after the extubation, use non-invasive ventilation or nasal cannula to inhale oxygen according to the patient's condition, and different types of respiratory failure are given different oxygen delivery methods [50]. In addition, COPD patients often compensate by increasing the number of breaths. This compensation is mostly chest breathing [51]. The effect of chest breathing is lower than that of abdominal breathing [52]. Patients will experience varying degrees of fatigue. Therefore, patients need to be encouraged to perform breathing exercises after extubation.

Due to various factors such as tracheal intubation, restraint, separation from relatives, anxiety and fear, etc., various degrees of pain should be weighted by the medical stuff. Pain makes the patient's stress response aggravated and a series of physical and psychological changes occur [53, 54]. Medical staff should pay attention to the impact of pain on delirium, conduct daily pain assessment, observe whether the patient has physical and psychological changes, and implement intervention measures as soon as possible to reduce the pain of the patient and prevent the occurrence of delirium [55]. For patients with pain, the evaluation of the location, accompanying symptoms, and duration of the pain, follow the doctor’s instructions for analgesia, and observe the effect of the medication [56, 57]. Besides, the medical stuff should adjust the dose of the drug according to the effect, avoid underdose or overdose. Underdose of analgesia drugs will not achieve the pain relief effect, and overdose will cause adverse reactions such as respiratory depression [58]. Therefore, it is particularly important to strengthen the evaluation of pain and analgesic effects of such patients.

Sedation is a commonly used treatment for patients with COPD and respiratory failure undergoing mechanical ventilation. Sedation can cause dysfunction of the craniocerebral nervous system, affect the response to stimuli, and induce delirium [59]. Commonly used clinical sedatives in ICU include propofol, dexmedetomidine hydrochloride, and midazolam. Among them, propofol can affect brain tissue perfusion and easily cause hypotension and respiratory depression [60]. Common adverse reactions of midazolam include drowsiness, headache, ataxia, hallucinations, etc., which can prolong the length of ICU hospitalization and mechanical ventilation, and increase the incidence of delirium [61]. If the drug is stopped suddenly, it will cause withdrawal symptoms, so the dose should be gradually reduced, and the benzodiazepine antagonist flumazenil should be used to reverse the overdose [62]. Dexmedetomidine may be less likely to cause delirium, than other sedative agents in the ICU [63–65]. Dexmedetomidine hydrochloride has a mild sedative effect and is suitable for mechanically ventilated patients. Patients are easy to wake up but easily cause hypotension [66]. in addition, dexmedetomidine hydrochloride increases the stimulation of the vagus nerve and can cause bradycardia [67]. Previous studies [68, 69] have pointed out that dexmedetomidine hydrochloride has a good sedative effect on mechanically ventilated patients, which can reduce the incidence of delirium and shorten the duration of delirium. Daily sedation interruptions should be implemented for sedated patients to avoid excessive use of sedative drugs, reduce drug accumulation, and facilitate early extubation of patients [70].

Several limitations of this present study must be considered. Firstly, sedation is an important factor of development of delirium, sedation is not commonly performed in our study, and the drugs used for sedation in this study are different, the associations of delirium and sedation drug use need more investigations in the future with larger sample size. Secondly, we do not have the data on the severity of COPD at admission, which may be associated with the occurrence of delirium. It’s necessary to evaluate the association of COPD severity and delirium in the future. Thirdly, this is a single-center study, and its generalizability may therefore be limited. Even the sample size has been calculated for statistical power to detect the group differences, future studies with muti-centers and larger sample size are needed in the future to further elucidate the influencing factors of delirium in COPD patients.

## Conclusions

In summary, this study has found that delirium in patients with COPD combined with respiratory failure undergoing mechanical ventilation is 21.94%, and age ≥ 75y, BMI ≤ 19 kg/m^2^, hypertension, APACHE II score ≥ 15, CPOT ≥ 5, sedation, PaO_2_ ≤ 75 mmHg are the risk factors of delirium. Therefore, medical staff should pay more attention to patients with risk factors and take intervention measures as soon as possible to reduce the incidence of delirium and avoid the occurrence of delirium-related adverse events. However, limited by sample size, more studies with comprehensive variables are needed to further evaluate the influencing factors of delirium, to provide insights to the clinical management and prevention of delirium in COPD patients.

## Data Availability

All data generated or analyzed during this study are included in this published article.

## Abbreviations

COPD: Chronic obstructive pulmonary disease
ICU: Intensive care unit
CAM-ICU: ICU confusion assessment scale
BMI: Body mass index
GCS: Glasgow coma scale
APACHE II: Acute physiology and chronic health score
CPOT: Critical pain observation tool
T3: Triiodothyronine
T4: Thyroxine
TSH: Thyroid stimulating hormone
FT3: Free triiodothyronine
FT4: Free thyroxine
PaO_2_: Arterial partial pressure of oxygen
PaCO_2_: Arterial partial pressure of carbon dioxide
